# Mechanical and Compositional Implications of Gallium Ion Milling on Epoxy Resin

**DOI:** 10.3390/polym13162640

**Published:** 2021-08-08

**Authors:** Raz Samira, Atzmon Vakahi, Rami Eliasy, Dov Sherman, Noa Lachman

**Affiliations:** 1 Department of Materials Science and Engineering, Tel Aviv University, Tel Aviv 6997801, Israel; dovsherman@tauex.tau.ac.il; 2 Center for Nanoscience and Nanotechnology, The Hebrew University, Jerusalem 9190401, Israel; atzmonv@savion.huji.ac.il; 3 School of Mechanical Engineering, Tel Aviv University, Tel Aviv 6997801, Israel; eliasy@tauex.tau.ac.il

**Keywords:** in situ deformation, transmission electron microscope, irradiation effects, mechanical properties, epoxy

## Abstract

Focused Ion Beam (FIB) is one of the most common methods for nanodevice fabrication. However, its implications on mechanical properties of polymers have only been speculated. In the current study, we demonstrated flexural bending of FIB-milled epoxy nanobeam, examined in situ under a transmission electron microscope (TEM). Controllable displacement was applied, while real-time TEM videos were gathered to produce morphological data. EDS and EELS were used to characterize the compositions of the resultant structure, and a computational model was used, together with the quantitative results of the in situ bending, to mechanically characterize the effect of Ga^+^ ions irradiation. The damaged layer was measured at 30 nm, with high content of gallium (40%). Examination of the fracture revealed crack propagation within the elastic region and rapid crack growth up to fracture, attesting to enhanced brittleness. Importantly, the nanoscale epoxy exhibited a robust increase in flexural strength, associated with chemical tempering and ion-induced peening effects, stiffening the outer surface. Young’s modulus of the stiffened layer was calculated via the finite element analysis (FEA) simulation, according to the measurement of 30 nm thickness in the STEM and resulted in a modulus range of 30–100 GPa. The current findings, now established in direct measurements, pave the way to improved applications of polymers in nanoscale devices to include soft materials, such as polymer-based composites and biological samples.

## 1. Introduction

With the increased demand for micro- and nano-scale devices, such as micro-electromechanical systems (MEMS), more development is put into the study of the mechanical properties of materials in decreased length-scales. To promote these studies, advanced small-scale mechanical testing methods are used, which can isolate features of interest in the sample and measure them separately. For example, increased plasticity and strength was demonstrated by compression of nanometric metallic pillars [1,2,3,4,5,6], tension of nanowires and films [7,8,9,10], and bending of microbeams [11,12].

A powerful method for investigating the mechanical properties of small-scale specimens is by quantitative mechanical testing in situ Transmission Electron Microscope (TEM). This method allows a simultaneous collection of mechanical data with real-time structural images, thereby connecting material structure and properties. However, the fabrication of such nanometric specimens presents a great challenge, especially when specific mechanical testing geometries are required, without modifying the composition and morphology of the material [9,13,14,15,16,17,18]. One of the most prevalent methods for nanometric samples’ fabrication is the focus ion beam (FIB) milling, most commonly with a gallium ion source (Ga^+^).

Removal of material by ion milling can lead to several ion irradiation effects on the material, which might change the microstructures and consequently the mechanical properties of samples [9,13,14,15,16,17,18]. Studies were mainly conducted on metallic and semiconductor materials, and have revealed that Ga^+^ ion implantation occurs in depth of up to several tens of nanometers, depending on the kinetic energy of the beam (acceleration voltage) and the incidence angle of the used ions, and on the milling geometry [16,19,20,21,22]. The main implication is defects at the layer adjacent to the milled surface, including dislocations, amorphization, and intermetallic formation [16,21,23]. This has shown ambivalent outcomes on the mechanical performance, since the damaged layer sometimes reported as showing an increase in strength and hardness [9,16,17,18], but exhibiting decreases in other cases [13,15].

In polymeric materials, the ion irradiation damage is even more severe. It has been shown that the Ga^+^ ions are implanted even deeper into the material, since carbon atoms are lighter [22,24]. There are also local thermal effects, which can cause degradation of the polymer or rapid melting of the material [7]. The molecular structure can also be affected by the ion beam [25,26] or even by the electron beam of the electron microscope [27,28]. This damage is considered unavoidable using electrons microscopy (FIB included); however, previous studies have shown that lower currents and exposure time in the final thinning steps can reduce thermal effects and roughening of the surface [7,29]. Lowering the beam energies can decrease the thickness of the damaged layer but compromise the spatial resolution because the beam is wider [30]. This resolution reduction presents a problem when trying to shape small and specific geometries.

Only few studies have used FIB to fabricate small-scale polymeric specimens for mechanical testing, to study the effects of size on mechanical behavior [25,31,32,33]. Moon demonstrated patterns of wrinkled stiff skin on a polymeric substrate upon exposure to FIB [33]. Wang performed micro compression experiment on FIB-milled epoxy micropillars and concluded that a non-negligible stiff surface skin is created, and estimated a Young’s Modulus of 30 GPa and a thickness of 30 nm [32]. Nathan demonstrated a tensile test of polymethylmethacrylate (PMMA) under an optical microscope and in situ TEM [31]. They highlighted the sensitivity of polymeric materials to the TEM electron beam and to the radiation effects by the FIB. The important conclusion is that the Ga^+^ ions are embedded within the material surface and cause a stiffening effect. FIB milling with helium ions is proposed as a way to counter the effect of Ga^+^ FIB milling, as it produces much sharper edges with significantly less ion implantation [22]. The much lighter ions undergo smaller lateral scatter and have a stopping distance far greater than the film thickness. However, the helium ion milling rate is much slower than gallium milling, fitting only for small volumes.

However, none of the above-mentioned studies quantify, or even validated, the morphological and compositional changes caused by the ion beam effect on the mechanical properties of polymers. As polymers are increasingly used in MEMS as an alternative to conventional materials such as silicon and metals, it is crucial to evaluate their mechanical response to irradiation, as a way to control and perhaps even to enhance device performance [34,35,36]. We propose a quantitative mechanical testing in situ TEM, which allows a simultaneous collection of mechanical data with real-time structural images. Together with finite-element analysis, the powerful combination allows for quantitative adjustment of predicted properties to measured results, thereby connecting material structure and properties.

Using an in situ TEM bending test, the current study used Ga^+^ ion FIB milling to fabricate epoxy resins’ nanoscale samples and characterized the mechanism by which irradiation damage affected mechanical failure. The epoxy nano-cantilevers were bent with a sharp diamond tip until crack initiation; the crack propagation was recorded, and post-fracture cracks were carefully examined. Finite element analysis (FEA) was used to predict and validate the experimental results. The irradiation damage was evaluated with a Scanning Transmission Electron Microscope (STEM), combing high-angle annular dark-field imaging (HAADF), energy-dispersive X-ray spectroscopy (EDS), and electron energy loss spectroscopy (EELS) methods.

## 2. Materials and Methods

**Figure 1:**
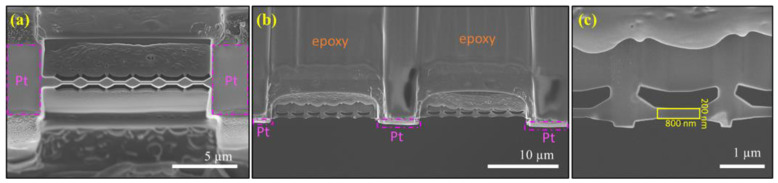
SEM images of dual-clipped cantilever fabricated with FIB; (**a**) top view; (**b**) side view series of beams. Protective Pt layer is deposited on the surface of the epoxy; (**c**) side view (the specimen is marked by yellow rectangular), length = 800 nm; width = 200 nm; thickness = 200 nm.

Bulk samples of a two-component aerospace-grade epoxy compound (EP 520/EPC 520; Polymer-G, Gvulot, Israel) were made according to the manufacturer’s instructions (see Supplementary Materials). Aerospace-grade epoxies are commonly used in structural materials as composite matrices, such as carbon/epoxy or glass/epoxy composites. The epoxy provides excellent mechanical properties and environmental resistance at a low weight. Separate samples were used for the bulk mechanical tests and the fabrication of the nano samples. A small piece was precisely cut from the bulk, using an automatic dicing saw (Disco DAD 3350, Disco Corporation, Tokyo, Japan), in the dimensions of 1800 × 1500 µm^2^ (length × width). A thin lamella of 50 µm thickness was then micromachined, ready for Focused Ion Beam (FIB) milling. Subsequently, double-clamped cantilever structures were cut by a gallium FIB at 30 kV ion acceleration voltage (FEI Helios NanoLab 460F1 dual-beam FIB-SEM, FEI Company, Hillsboro, OR, USA). A thin layer of iridium (~2 nm) was sputtered (Quorum Q150V S Plus Sputter Coater, Quorum Technologies, Laughton, UK) on the sample surface prior to insertion to the FIB, to improve the conductivity inside the electron microscope. The fabricated cantilevers at the end of the process are hereby presented (Figure 1; dimensions of l:w:t are 800 × 200 × 200 nm^3^, respectively). The full fabrication process is described in the Supplementary Materials (Figures S1 and S2).

Brittle materials are favorably tested in bending mode compared to tension, since the specimen clamping required in tensile tests often causes premature failure. Therefore, the dual-clipped cantilever configuration was chosen for the bending test. The FIB process started with electron-assisted (200 nm thick) and ion-assisted (800 nm thick) deposition of a platinum strip on the top surface of the area of interest to prevent ion-beam damage and unwanted sputtering. Subsequently, an electron-transparent lamella 200 nm thick with parallel surfaces was shaped (Figure 1a). We then milled a cut perpendicular to the bending direction to form the cantilever shapes (Figure 1b,c).

The epoxy cantilevers were tested in a FEI Tencai 20 TEM (Hillsboro, OR, USA), coupled with Bruker (Hysitron, Minneapolis, MN, USA) PicoIndenter 95 (PI-95) TEM holder. The TEM was operated at 200 keV, in bright-field mode and an objective lens of 60 µm to increase contrast. To reduce possible electron beam damage, a 100 µm condenser aperture was used to reduce the electron count, and exposure times on the samples were kept to minimum. A total of 10 cantilevers were bent with a sharp wedge diamond tip of approximately 50 nm radius of curvature. The force was applied perpendicular to the cantilevers, in displacement-controlled mode, at a displacement rate of 5 nm/s, and included 200 data points per second of force and normal displacement. Videos were recorded using digital capture of the Gatan One View camera (Gatan Inc., Pleasanton, CA, USA) at eight frames per second and 4k resolution. The load-displacement data and the real-time video were recorded and synchronized using the frame grabber feature of the TriboScan software (Hysitron, Minneapolis, MN, USA). The samples were deformed in one cycle until catastrophic failure occurred. Post-fracture images were taken with high magnification for further analysis of the resulting crack.

Bulk cantilever epoxy specimens (l:w:t are 50 × 12.5 × 1 mm^3^, respectively) were bent in a mechanical testing system (Instron, Norwood, MA, USA), equipped with 1 kN load cell and 3-point bending clamps. The experiments ran in displacement-controlled mode at 2 mm/min crosshead speed.

The nanometric test geometries were modeled using FEA (Abaqus, version 2020, Johnston, RI, USA) to validate the experimental results and study the evolution of the stress and deformation in the samples. The finite element mesh consisted of 14,755 eight-noded biquadratic elements with reduced integration (CPS8R) and 48,702 nodal points. Plane stress conditions were assumed, and a linear static stress analysis was performed. The material properties of the epoxy were set to the values of the bulk material: 3.5 GPa for Young’s modulus and 0.3 for Poisson’s ratio. Young’s modulus of the FIB-induced stiff layer was calculated by the simulation via fitting the simulated force-displacement curve to the experimental results. The modulus values were set to 30, 37, 47, and 100 GPa and Poisson’s ratio to 0.3.

New TEM samples were fabricated by FIB to evaluate the ion irradiation damage on the epoxy, by intentionally bombarding the surface with Ga^+^ ion beam at 30 kV in a shape of rectangular (see Supplementary Materials for the full process; Figure S3). The samples were then covered with a protection layer of platinum. The lamella was lifted and attached to a TEM grid and finally thinned to an electron transparent thickness (70 nm). The samples were then imaged in an Aberration Probe-Corrected Scanning Transmission Electron Microscope Themis Z G3 (ThermoFisher, Waltham, MA, USA). The STEM was operated at 80 kV, imaging with High-Angle Annular Dark-Field (HAADF) detector. Energy-dispersive X-ray Spectroscopy (EDS) and electron energy loss spectroscopy (EELS) methods (Enfinium spectrometer, ER977, GATAN Inc., Pleasanton, CA, USA) were also used to provide an elemental mapping, importantly showing the Ga^+^ ion implantation, content percentage, and the thickness of the affected layer.

## 3. Results and Discussion

**Figure 2:**
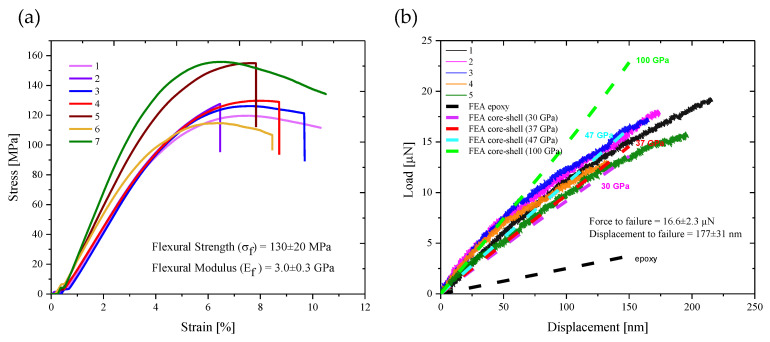
Flexural bending results. (**a**) Stress-strain curves of macroscopic specimens (50 × 12.5 × 1 mm^3^, length × width × thickness). Average flexural strength (UTS) σ_f_ = 130 ± 20 MPa, flexural modulus E_f_ = 3.0 ± 0.3 GPa; (**b**) nanoscale bending test results of five representative epoxy specimens. From the load-displacement curves, the average load at failure was 16.6 ± 2.3 µN, and average displacement at failure was 177 ± 31 nm. The FEA simulation results of pure epoxy and ‘core-shell composite’ with four different modulus values of the shell (30, 37, 47, and 100 GPa) are represented by the dashed lines.

The in situ experiments enabled visualization of the onset of deformation and fracture, while directly correlating them to “pop-in” events (sudden displacement bursts) in the load-displacement curve. In metallurgic research, fractures are mainly carried by dislocations, which can be traced by the TEM during the deformation process. However, amorphous materials such as thermoset polymers do not contain crystalline domains, and therefore no dislocations can be visualized. TEM in these materials is thus used to track the flow of stresses across the beam by changes in image contrast, and the propagation and trajectory of cracks. Initial mechanical properties of the epoxy were gathered by flexural bending of the macroscopic samples (Figure 2a). The mechanical behavior of the irradiated samples was gathered by flexural bending in situ the TEM (Figure 2b). The stress and strain of the macroscopic samples were calculated from the Euler–Bernoulli beam theory [37].

Since our nano-scaled geometries did not follow Euler–Bernoulli beam theory assumptions (as they are shorter than the standard 1:10 thickness to length ratio, in order to fit the TEM detection frame), we could not directly calculate the stress and strains. Thus, we performed an FEA to validate the analytical results, and assess the failure stress in our specimens (dash lines in Figure 2b, to be further elaborated later on). The macroscopic cantilever specimens exhibited visco-elastic deformations (Figure 2a) while the nanoscale cantilevers displayed a linear elastic deformation (Figure 2b) up to brittle fracture. The brittle-like failure was apparent in a sudden drop in load, characteristic of isotropic and amorphous polymers such as epoxy crosslinked with amine hardeners. Since the testing temperature was well below the glass transition temperature (T_g_ = 119 °C, as measured by DSC 250 Differential Scanning Calorimeter, TA Instruments, New Castle, DE, USA), it supports the observed hard and brittle mechanical behavior.

The experimental geometry was modeled in FEA to investigate the elastic stress at crack onset and to validate the experimental results. Initially, we modeled a pure epoxy cantilever with Young’s modulus of 3.5 GPa and Poisson’s ratio of 0.3 (according to the manufacture data, and to literature, as polymers do not show true nanoscale effects when the specimens are thicker than 50 atoms [38]). The simulated force-displacement curve (Figure 2b, ‘FEA epoxy’) showed significantly lower force values compared with the experimental results. It should be noted that FIB damage as inclusions, Ga^+^ ions’ damage, or Pt residues was not initially visible in the SEM images (Figure 1). Nevertheless, the ion beams used in FIB are known to modify the targeted surface by atomistic collision cascades, developing residual strain and stiffening of the surface layer, effectively peening the material [25,32,33,39], with polymers more prone to be affected. Particularly, Wang et al. [32], using diglycidyl ether of bisphenol A (DGEBA) and piperidine (cyclic amine), equivalent to the one studied here, proposed the core-shell model, stating that the epoxy specimens could be regarded as a ‘composite’ material, consisting of an epoxy core surrounded by an FIB peening-induced stiff layer. Similar stiffening effects of the surface was reported by Nathan for a PMMA film in tension [31] and by Schamel for epoxy resin pillars in compression [39]; the three studies used Ga^+^ FIB milling with an acceleration voltage of 30 kV.

**Figure 3:**
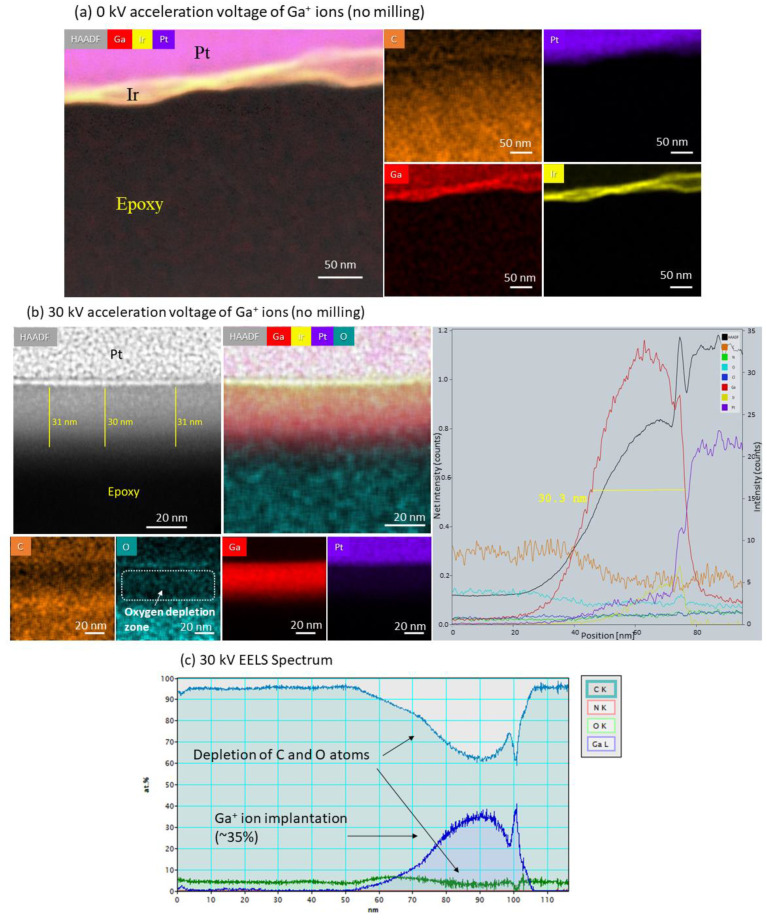
HAADF–STEM imaging and analysis of Ga^+^ ions damage on epoxy. (**a**) 0 kV acceleration voltage of Ga^+^ ions (no milling)—HAADF–STEM image and corresponding EDS elemental distribution map of iridium (yellow), platinum (purple), carbon (orange), and gallium (red). (**b**) 30 kV acceleration voltage of Ga^+^ ions—(**left**) HAADF–STEM image and corresponding EDS elemental distribution map of oxygen (blue), platinum (purple), carbon (orange), and gallium (red). Note the gallium layer thickness of 30 nm and depletion of oxygen in that layer. (**Right**) EDS spectrum of 30 kV—intensity counts of each element according to the position (nm). It also measures 30 nm thickness of gallium layer. (**c**) EELS results (line scan) of 30 kV—atomic percentage of carbon, gallium, oxygen, and nitrogen. The figure shows high implantation amount of Ga and a reduction in the atomic percentage of O and C in the same area. Note: the peak at 100 nm is artificial due to stitching of the spectrums.

As this core-shell model could explain the mechanical observations, we created a new FE model for the ‘composite’ material (colored dash lines in Figure 2b). However, initially, the ion beam irradiation damage had to be evaluated; hence, new TEM samples were fabricated with FIB by intentionally damaging the surface with Ga^+^ ions and then imaged in STEM (Figure 3 and Figure S4). Four samples were fabricated and compared—unmilled (Figure 3a—‘0 kV’) and milled, with an acceleration voltage of 8 kV (Figure S5) and 30 kV (Figure 3b—‘30 kV’). With the HAADF detector coupled with the EDS technique, the elements in the sample could be mapped, and the damaged layer could be identified. The unmilled sample images show evidence of the initially sputtered Ir (yellow) on the surface with the protective Pt (purple) layer covering it. Although the sample was not milled with Ga, it is still present in the sample. This implies that even if Ga is not directly exposed to a region, it can still be sputtered from a nearby milling region. In the 30 kV milled sample, the damaged layer is easily seen and is measured at 30 nm thickness. The Ga layer is concentrated at the surface, and a distinct Pt layer covers it. The thickness of the layer also measures at 30 nm in the EDS spectrum (Figure 3c). To complement the EDS measurement, we also performed an EELS measurement (Figure 3c) because it is more sensitive to light atoms than the EDS technique. The data were gathered via a line scan, which showed a region of epoxy between 0 and 65 nm; then, the Ga atoms started to appear at 60 nm up to 105 nm, and beyond was the Pt layer. The spectrum shows a high content of Ga implanted, almost 40%, at the range of 70–100 nm, which confirms the 30 nm thickness from the EDS measurement, and in previous studies [22,32,39]. The high content of Ga, accompanied with the decrease in the C content, indicates an extensive sputtering of C atoms from the surface and a considerable implantation of Ga atoms. Furthermore, the EDS and EELS point at decreasing content of O in the damaged layer, which indicates structural change of the epoxy. The mechanism is explained by Possart et al., demonstrating that oxygen content is depleted by radical formation due to ionization, dehydrating the epoxy to form an alkene linkage [40]. This high concentration of Ga can explain the observed stiffening effect on the surface of the epoxy. While the epoxy is prone to change chemically by the electron and ion beams [25,26,27,28], it is possible that the amorphous damage at the surface mainly contributes to the stiffening effect. As seen in the EELS spectrum, the big Ga^+^ ions are exchanged with the small carbon atoms, creating strong compression stresses at the surface, which compensate for the applied tension. As a result, larger forces are needed to cause failure, as seen in the in situ stress-strain curves. Chemical tempering of glass exhibits similar strengthening mechanism in which small ions are exchanged with bigger ones, causing a rise in the residual compressive stress state below the surface [41,42].

**Figure 4:**
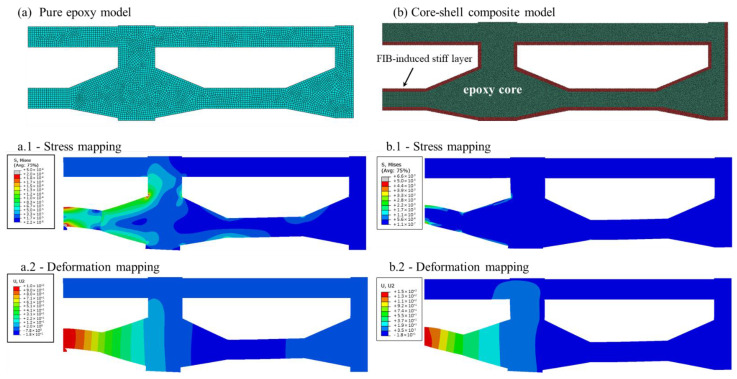
Finite Element Analysis of the cantilever beam during loading: the far right and top of the specimen were subjected to fixed under-boundary conditions (y and x only). The load was applied through a pin at the far bottom left of the model. (**a**) Pure epoxy model. (**a.1**) Stress contour plot and (**a.2**) deformation contour plot for the epoxy; maximum deformation of 150 nm and maximum stress of 183 MPa to occur at the point opposite to the loading point. (**b**) Core-shell composite model. (**b.1**) Stress contour plot and (**b.2**) deformation contour plot for the core-shell composite model; maximum deformation of 150 nm and maximum stress of 2.8 GPa to occur at the point opposite to the loading point. Stresses are higher at the stiff shell of 30 nm thickness. Due to symmetry, only half of the entire 3 beam model was analyzed.

According to the acquired information, a new FE model was designed for a core-shell composite (Figure 4b). The model consists of an epoxy core with Young’s modulus of 3.5 GPa, and a stiff layer of 30 nm thickness (about 30% of the volume of the specimen, making Ga^+^ effect more prominent the thinner the specimen). Various Young’s moduli were modeled by the simulation, via fitting the simulated force-displacement curve to the experimental results (Figure 2b, ‘FEA core-shell composite (37 GPa)’). The value of 37 GPa—our initial estimation—fits between the calculations of Schamel [39] (who calculated a stiff layer of 20 nm and 47 GPa; our calculations—Figure 2b, ‘FEA core-shell composite (47 GPa)’—show that, while the thickness might be too thin, the modulus still fits the experimental results) and Wang [32] (who calculated a stiff layer of 30 nm and 30 GPa; our calculations—Figure 2b, ‘FEA core-shell composite (30 GPa)’—indicating that this modulus estimation is slightly an undershooting); thus, validating their calculations with measurements. An overshooting of 100 GPa modulus was also calculated, showing an agreement with the initial moduli of the experimental results. We thus conclude, from the combination of morphological characterization, experimental results, and FEA simulations, that the modulus of the chemically-tempered carbon layer is 30–100 GPa—similar to that of amorphous carbon [43].

The simulation applied a 150 nm displacement and mapped the deformations and stresses across the cantilever. The calculated stress at the surface where the crack initiated is 2.8 GPa (Figure 4(b.1)), which was extracted from the integration points’ stresses at small elements at the edge of the specimen, where the maximum stresses are generated. The stiff outer layer absorbs most of the applied stress, and only negligible stresses are transferred to the underlying epoxy core. The irradiation effects on the mechanical properties are evident in the significant increase in the ultimate strength of the nano-scaled samples.

**Figure 5:**
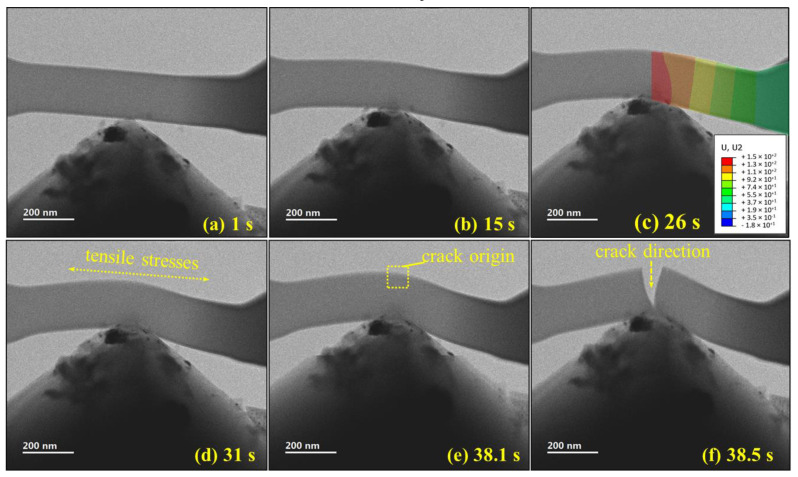
Continuous frames of representative in situ TEM bending test; bright field mode, 200 kV, objective aperture 60 μm. (**a**) Bending test after 1 s; (**b**) bending test after 15 s; (**c**) stacking of deformation contours according to the computational modeling, demonstrating a good fit to the observed deformation and thus another validation of the modeling assumptions; (**d**) tensile stress accumulating at the opposite side of the tip; (**e**) few frames prior to fracture; (**f**) rapid crack opening. The figures show nonsymmetric deformation due to misalignment.

The temporal evolution from the in situ experiment video of a representative sample was captured for failure analysis (Figure 5). The sample underwent loading at a constant deformation rate. Image contrast indicated stress movement across the beam, from the edges to the middle point of applied force. Under that load, bending moments produced tensile stresses at the top surface of the cantilever, initiating a single crack, which then propagated swiftly across the beam. The brittle, swift failure was accompanied with a sudden drop in force preceding the crack. The core-shell observed structure can also explain the fast crack propagation because once the hard shell fails, the core is too weak to arrest the crack. Brittle fracture was also reported in the studies by Wang and Schamel for the submicron irradiated epoxy pillars [32,39]. As the failure occurred too quickly for the camera to capture its progression and crack opening, it would be beneficial to reduce the video resolution (from 4k to 1080p) in order to increase the frame rate collection.

**Figure 6:**
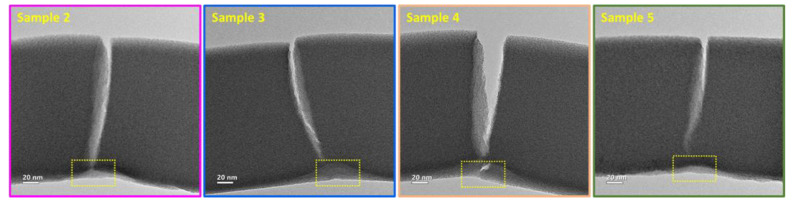
TEM images of fractured beams, after the in situ bending test. Bright field, 200 kV, objective aperture 60 μm. The post-fracture images show the trajectory of the crack propagation and indentation marks (highlighted by dashed yellow squares) caused by the sharp wedge of 50 nm tip radius.

High-magnification TEM images of the post-test fractured beams were used for post-failure analysis (Figure 6). The video and the images (Figure 5 and Figure 6) demonstrate a mode I fracture and crack propagation by opening in the direction of the maximum tensile stress toward the loading line. Small (a few nanometers) deviations from sample center in loading were shown to divert the crack propagation trajectory to the opposite side of the loading line (Figure 6, samples 3 and 5). However, when the nanoindenter was better-positioned, the cracks followed a straight path (Figure 6, samples 2 and 4). Indentation marks are visible at the bottom of the beam, where the force was applied (Figure 5, highlighted by dashed yellow squares).

## 4. Conclusions

To summarize, the effects of Ga^+^ FIB irradiation on the microstructure and mechanical properties of scaled down epoxy resin were investigated utilizing an in situ TEM bending test, HAADF–STEM, and a finite element simulation. First and foremost, great care should be taken when interpreting results from the FIB-milled polymeric specimens. The ion beam induces a cleavage of polymeric bonds and implements a high concentration of gallium on the outer surface, generating a stiff layer. Hence, the tested material could no longer be considered pure epoxy. The damaged layer was investigated in STEM and was measured at 30 nm, with high content of gallium (40%). The big Ga^+^ ions are exchanged with the small carbon atoms, creating strong compression stresses at the surface, consequently hardening the surface. Young’s modulus of the stiffened layer was calculated via the FEA simulation, according to the measurement of 30 nm thickness in the STEM and the mechanical in situ measurements and resulted in a modulus range of 30–100 GPa. The nanoscale epoxy sample displayed elastic behavior and a brittle fracture. The TEM in situ method, modified to fit polymers and other amorphous soft materials, combines a quantitative analysis and real-time structural images during failure in even lower length scales. This combination enabled the direct quantification of Ga^+^ milling on the mechanical properties of the sample. As polymers are increasingly used in miniaturized devices, it is crucial to evaluate their mechanical response and microstructural effects under the FIB technique. Moreover, a precise characterization of the FIB effect on polymers opens a possibility of utilizing this effect as part of the design and fabrication, thereby increasing design flexibility of micro- and nanoscale polymer-based devices. The methods established in this paper can be expanded to characterize composite materials and elaborate on the role of interface in mechanical properties.

## Data Availability

The data presented in this study are available upon request from the corresponding author.

## Supplementary Materials

The following are available online at https://www.mdpi.com/article/10.3390/polym13162640/s1, Figure S1: Step one of the nanosamples fabrication process-micromachining of the bulk. Figure S2: Fabrication process of nano cantilevers utilizing FIB. Figure S3: Fabrication process of irradiated samples for STEM utilizing FIB. Figure S4: Low magnification STEM images of the 0 kV, 8 kV, 16 kV and 30 kV samples. Figure S5: Sample 8 kV acceleration voltage of Ga+ - HAADF-STEM imaging and corresponding EDS elemental distribution map.
