# Supplementary material for: Mechanical and Compositional Implications of Gallium Ion Milling on Epoxy Resin
Source: Polymers (Basel). 2021 Aug 8;13(16):2640. doi: 10.3390/polym13162640 (PMC8398473; doi:10.3390/polym13162640)
Supplement: Supplementary file 1 [file polymers-13-02640-s001.zip › polymers-1328691-supplementary.pdf]

Supplementary Materials: Mechanical Implications of Gallium Ion Milling on Polymeric Materials

Raz Samira[a,*], Atzmon Vakahi[b], Rami Eliasy[c], Dov Sherman[a,c] and Noa Lachman[a,*]

1. Bulk Samples Fabrication

Bulk samples of a two-component epoxy compound (EP 520/EPC 520; Polymer-G, Israel) were made according to the manufacturer's instructions. The resin and hardener were mixed in a glass vial by a weight ratio of 100:30, respectively. The mixture was stirred vigorously until a homogenous blend was achieved, was degassed for one hour inside a vacuum oven ($1x10^{-3}$ torr), and was then poured into a silicone mold ($50\times12.5\times1$ mm^3 l:w:t) for thermal curing. Curing time was 24 hours in room temperature followed by four additional hours of post-curing at 100°C.

2. Nanosamples Fabrication

a. Micromachining

The deformation experiments performed *in-situ* the TEM required a modification of the current fabrication techniques of miniaturized specimens. This fabrication process was designed to suit polymers and to meet both the geometrical restraints of the system and the mechanical geometries. The approach taken for the present experiments was to begin the sample fabrication by shaping a thin and tall wall from the bulk sample, utilizing a micromachining process with an automatic dicing saw (Disco DAD 3350). Figure S1a illustrates the micromachining process. Initially, the bulk sample was precisely cut into the desired dimensions of 1800×1500

μm² (length × width). Then the sample was grinded and polished into a smooth surface 500 μm thick. The sample dimensions were selected carefully to ensure exact fit on the PI-95 wedge mount (figure S1d) and be short enough in height to prevent blockage of the electron beam in the TEM stage (figure S1c). The sample was further micromachined in the automatic dicing saw, by repeated and overlapping cuts in parallel to the long axis of the sample (feed speed of 1mm/sec and 16k rpm). The cuts were done on the top-polished surface, only 100 μm deep, by nickel-sintered blade 110 μm wide and 6-8 μm diamond grit size (Kulicke & Sofa; model # u4560). Each cut removed 110 μm in width of material and was carried on both ends of the cube until a tall and thin lamella remained in the middle, ready for FIB milling (Figure S1b).

It is beneficial to work directly on the bulk since the relatively large piece can be maneuvered easily and one piece can host hundreds of geometries, ready for indentation. It should be noted that this process is especially modified for polymers, since traditional micromachining methods, such as electrochemical etching, are only suitable for metals and ceramics. Selective etching is impossible since polymers are inert to most etching agents, and cutting methods need to be extra delicate and precise to fit the low hardness and amorphic structure of polymers. Figure S1c illustrates the sample configuration inside the TEM, relative to the electron beam and the indentation tip.

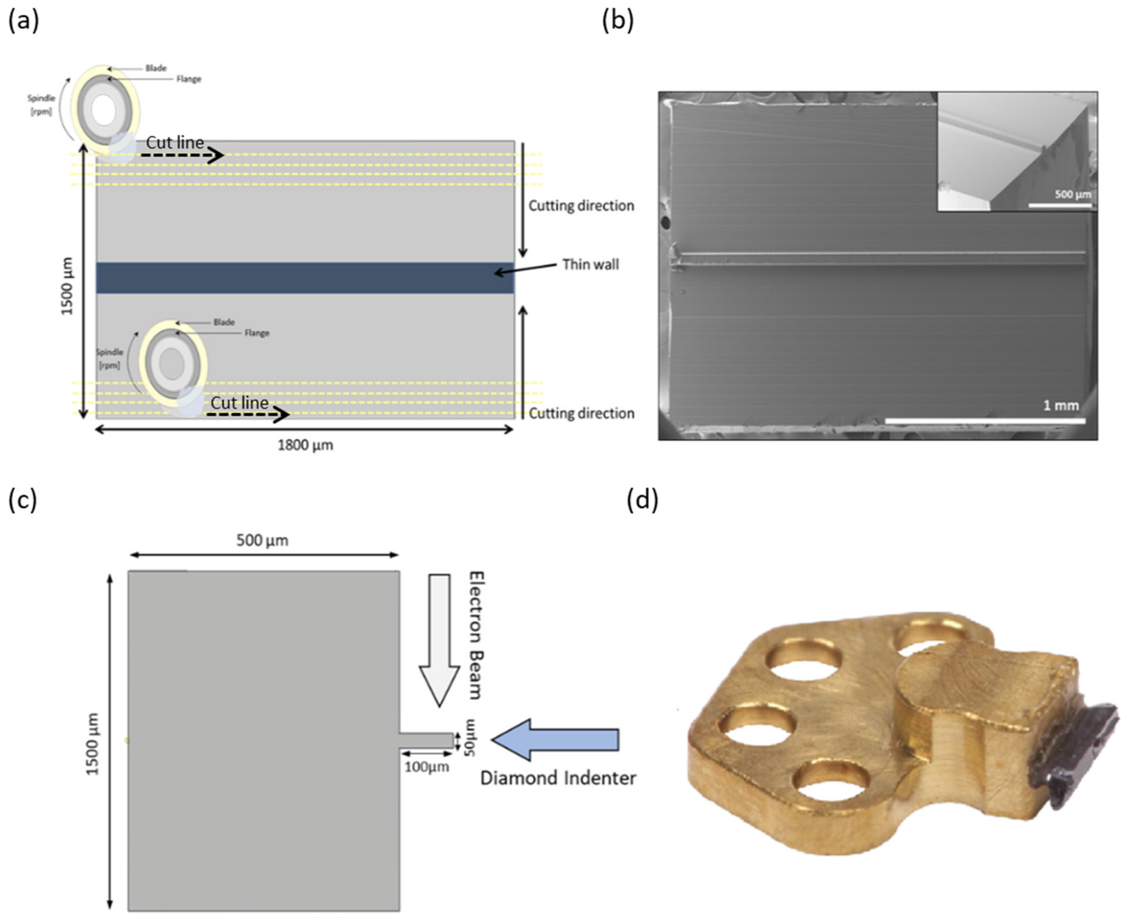

Figure S1. Step one of the nanosamples fabrication process - micromachining of the bulk. (a) illustration of the dicing process – machining a thin wall on the sample surface by repeated and overlapping cuts; (b) SEM image of epoxy wedge structure prior to FIB milling (top view. inset - tilted view); height=100 μm, width=50 μm; (c) illustration of the side-view of the sample after machining, ready for FIB milling; (d) PI-95 wedge mount configuration.

b. Focused Ion Beam (FIB)

FEI Helios NanoLab 460F1 dual-beam FIB-SEM system was utilized. Gallium ion beam energy remained constant at 30 kV for the entire process, but currents ranged from 47 nA to 24 pA, with the lower currents applied in the final steps to minimize FIB-induced damage. Prior to inserting the sample into the FIB, thin layer of iridium (~2 nm) was sputtered on the sample surface to improve the conductivity. The milling process was gradual to protect the structural integrity of the specimen.

Initially, a platinum strip was deposited (approximately 1 μm thick) on the top surface of the area of interest to prevent ion-beam damage and unwanted sputtering. Then, a wide window was milled of 110 μm length and 12 μm width, current was set to 47 nA (figure S2a). Next, 6 thinner windows were milled of 12.5 μm length and 4.5 μm width, with current set to 9.3 nA (figure S2b). The windows were further thinned to 800 nm thickness, current was set to 2.5 nA (figure S2c). Each window was designed to contain 6 cantilevers (figure S2d). Material was removed gradually, until electron transparent lamellas (<200 nm), with parallel surfaces were created; current was set to 40 pA (figure S2e). In this process, fresh surfaces that were undamaged by any possible structural defects and inclusions that might have originated in the micromachining step were discovered. The milling direction during the thinning was in parallel to the long axis of the lamella, to minimize the exposure of the surface to

the ion beam. The cantilever fixed ends were fabricated at 30° relative to the horizontal axis to ensure that the crack would propagate in the middle of the beam while bending. Subsequently, the sample was evacuated from the FIB chamber, tilted in 90° to its original orientation, and was inserted again. The thin lamellas were milled perpendicular to the top surface to form the double-clamped cantilever shapes, in dimensions of 800×200×200 nm^3 (l:w:t); current was set to 24 pA (figure S2f). This step inserted irradiation damage to the sample, as seen in the STEM analysis, since the

surface is directly exposed; however, it is a necessary step to form the cantilever shape. The protective platinum top-layer was removed at this stage.

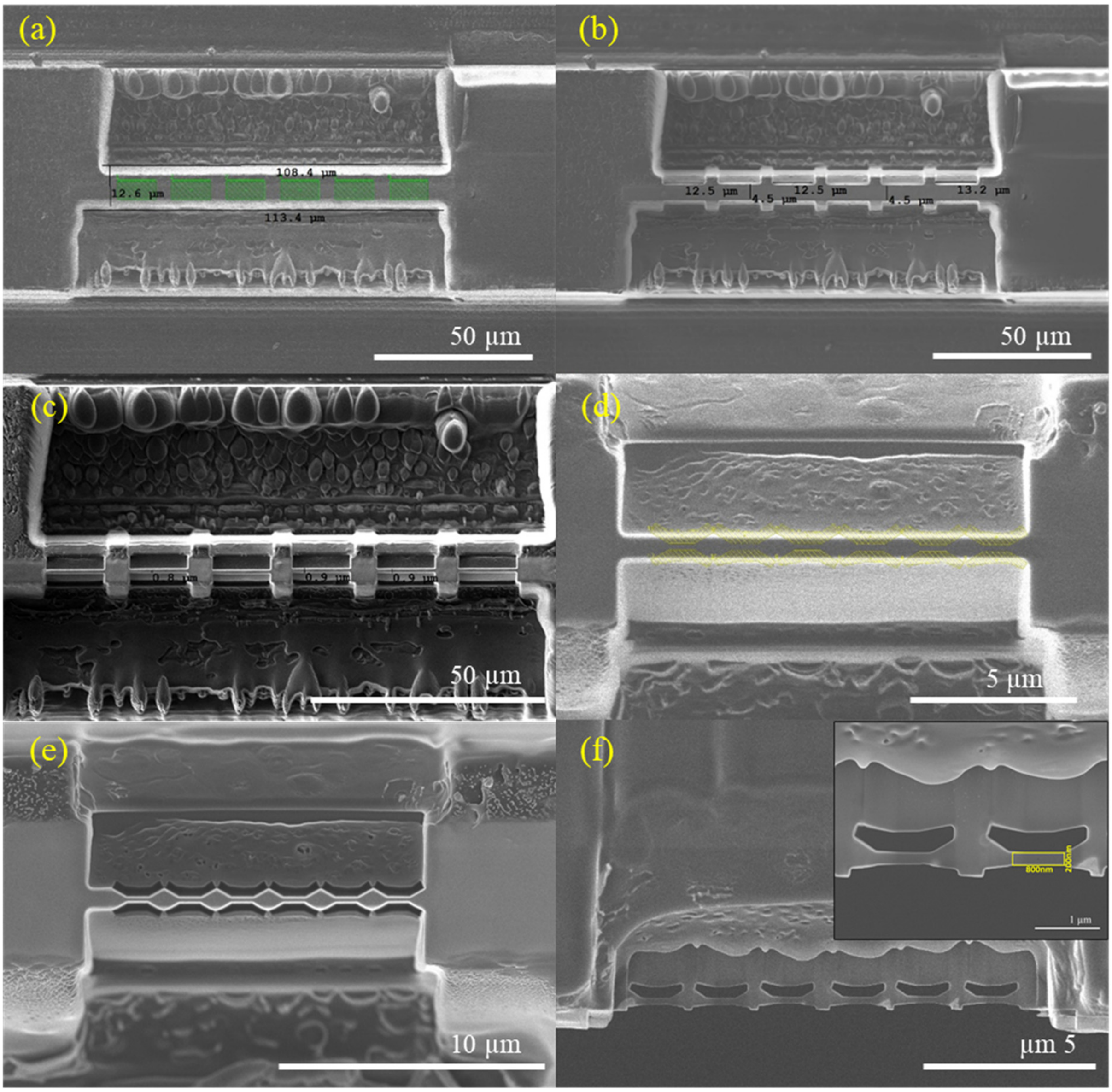

Figure S2. Fabrication process of nano cantilevers utilizing FIB. SEM images of: (a) wide window of 110 µm length and 12 µm width, current set to 47 nA; (b) 6 windows of 12.5 µm length and 4.5 µm width, current set to 9.3 nA; (c) windows further thinned to 800 nm thickness, current set to 2.5 nA; (d) the design of 6 cantilevers on a single window; (e) electron transparent lamellas (<200 nm) with parallel surfaces, current set to 40 pA; (f) 90° view of the resultant double-clamped cantilever, current set to 24

pA; in the inset a higher magnification of the cantilevers, in dimensions of 800×200×200 nm^3 (l:w:t).

3. STEM Analysis - Irradiated Samples Fabrication

New TEM samples were fabricated by FIB to evaluate the ion irradiation damage on the epoxy, by intentionally bombarding the top surface with Ga^+ ion beam. FEI Helios NanoLab 460F1 dual-beam FIB-SEM system was utilized. Prior to inserting the sample into the FIB, thin layer of iridium was sputtered on the sample surface to improve the conductivity. The process started with milling of 4 rectangles, 100 nm deep, each was bombard with different acceleration voltage of Ga^+ ions – 0 kV (no milling), 8 kV, 16 kV, 30 kV (figure S3a). The current was set to 24 pA as to mimic the induced damage done in the final step of the fabrication of the cantilevers (figure S2f). The area of interest was covered with a line of 200 nm electro-platina to prevent further damage and to delineate the location of the rectangles (figure S3b). Then, large trenches were milled surrounding the area of interest, using beam energy of 30 kV and 9.3 nA current (figure S3c). The lamella was then lifted with the FIB nanomanipulator, landed on a standard TEM grid, and fixed with carbon deposition (figure S3d). The lamella was covered with an additional protective layer of 1 μm ion-platina. The areas of interest were then thinned into TEM transparent thickness, initially by milling in 30 kV and 9.3 nA current from both sides of the lamella. The final thinning was carried with 5 kV and 24 pA to reduce damage on the sides of the lamella (figure S3d). Figure S3e illustrates the ion beam damage between the two

platina layers. The irradiation damage on the epoxy could only be seen in the STEM

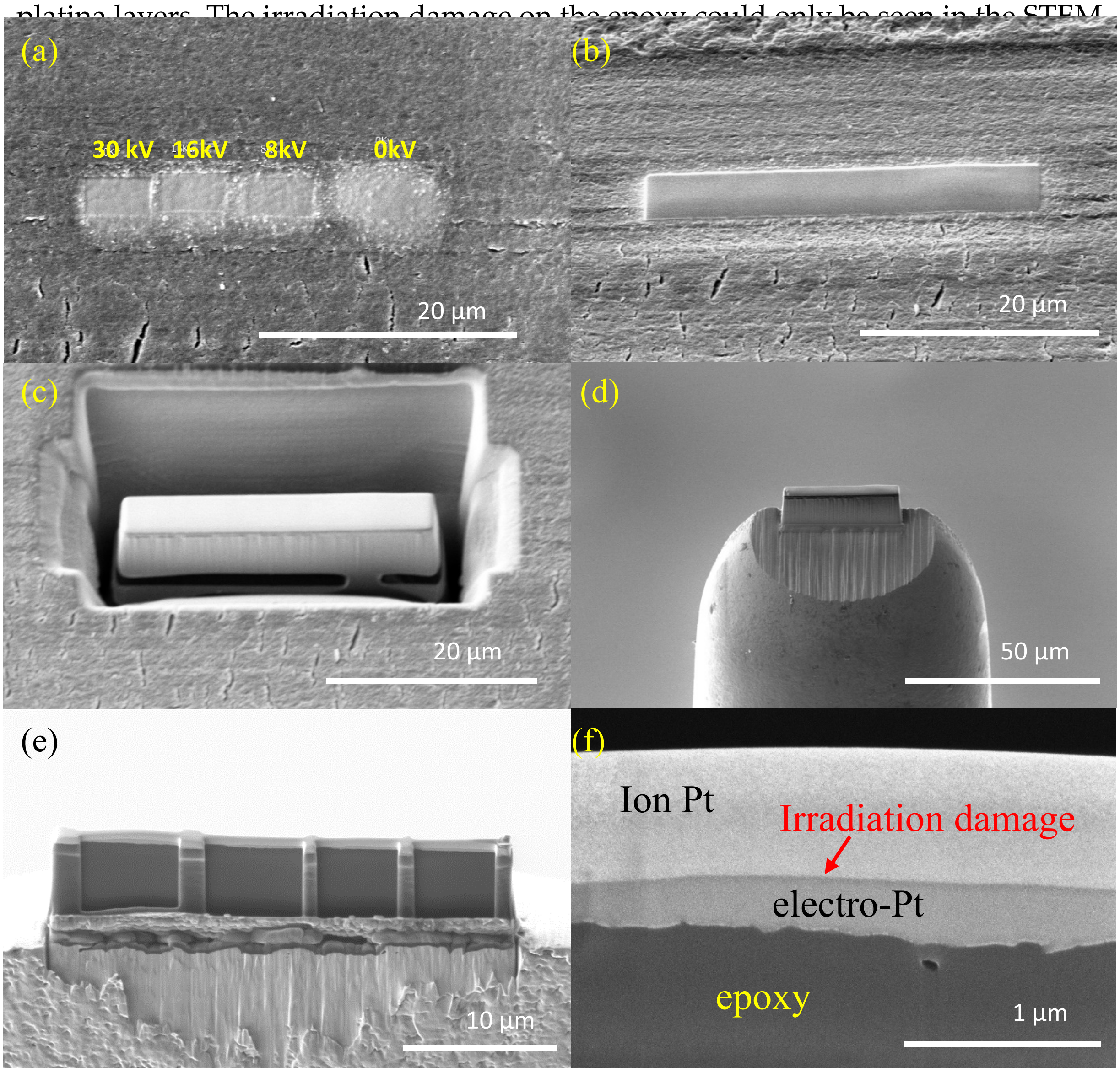

Figure S3. Fabrication process of irradiated samples for STEM utilizing FIB**.** SEM images of: (a) 4 rectangles, 100 nm deep, bombard with different acceleration voltage of Ga^{+} ions – 0 kV (no milling), 8 kV, 16 kV, 30 kV, current set to 24 pA; (b) area of interest covered with a line of 200 nm electro-platina; (c) large trenches milled surrounding the area of interest, beam energy of 30 kV and current set to 9.3 nA; (d) lamella fixed on a standard TEM grid with carbon deposition; (e) electron transparent lamellas with parallel surfaces, beam energy of 5 kV and current set to 24 pA; (f) demonstration of the ion beam damage between the two platina layers.

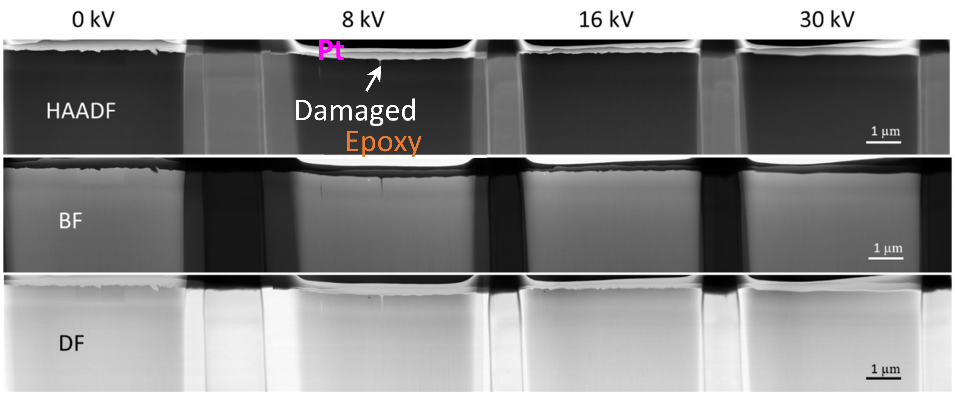

Figure S4. Low magnification STEM images of the 0 kV, 8 kV, 16 kV and 30 kV samples. The STEM was operated at 80 kV acceleration voltage and using the high-angle annular dark-field (HAADF), bright field (BF) and dark field (DF) detectors. The top layer is the protective Pt and underneath lays the epoxy. The thin irradiated layer is between the two layers.

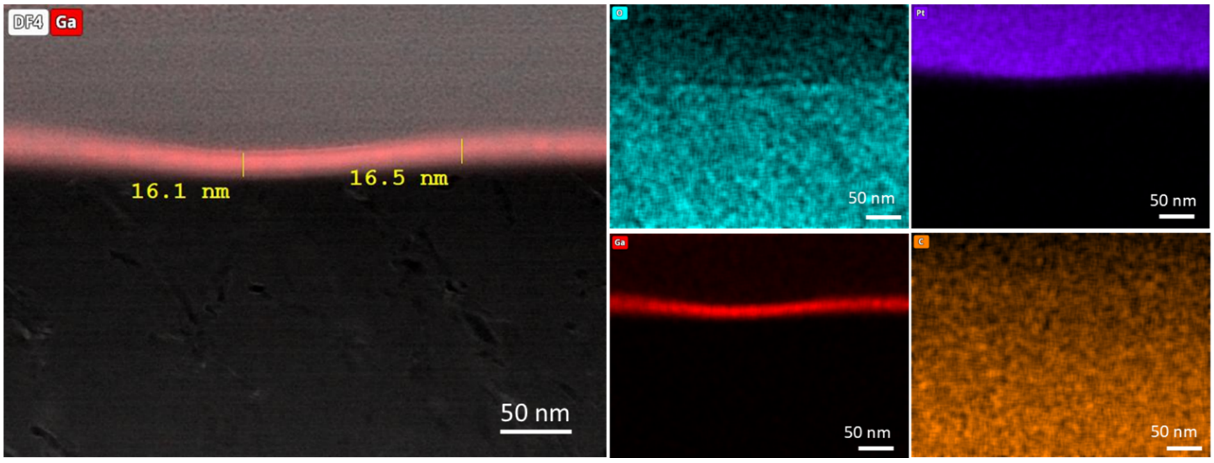

Figure S5. Sample 8 kV acceleration voltage of Ga^{+} - HAADF-STEM imaging and corresponding EDS elemental distribution map of oxygen (blue), platinum (purple), carbon (orange) and gallium (red). Note the gallium layer thickness of 16 nm.
